# Event-Based Vision Sensor Lifetime Degradation in Low Earth Orbit

**DOI:** 10.3390/s25216599

**Published:** 2025-10-27

**Authors:** Zachary Wilcox, Rui Graca, Brian McReynolds, John Williams, Saeed Afshar, Alexandre Marcireau, Matthew G. McHarg, Gregory Cohen

**Affiliations:** 1 International Centre for Neuromorphic Systems, Western Sydney University, Kingswood, NSW 2747, Australia; s.afshar@westernsydney.edu.au (S.A.); a.marcireau@westernsydney.edu.au (A.M.); g.cohen@westernsydney.edu.au (G.C.); 2 Institute of Neuroinformatics, University of Zurich and ETH Zurich, 8057 Zurich, Switzerland; 3 United States Air Force Academy Academy, 2345 Fairchild Dr., Colorado Springs, CO 80840, USAmatthew.mcharg@afacademy.af.edu (M.G.M.)

**Keywords:** low earth orbit, neuromorphic vision, noise, radiation, international space station, falcon neuro, falcon ODIN

## Abstract

We present the first study into the long-term effects of radiation on an Event-based Vision Sensor (EVS) using real-world data from orbit. Falcon Neuro is an experimental, first-of-its-kind payload attached to the exterior of the International Space Station (ISS) operating two DAVIS 240C Event-based Vision Sensors. This study considers data gathered by Falcon Neuro between January 2022 and September 2024 over a wide range of scenes from Earth-facing and space-facing sensors. Falcon Neuro contains the first working EVS system in orbit. While EVS radiation degradation has been studied on the ground, this is the first study of degradation for EVS cameras of any kind in a real, uncontrolled environment. EVS pixel circuits are unique, analog, and far more complex than CMOS or CCD cameras. By utilizing distinct and unique features in the data created by the different pixel circuits in the camera, we show that degradation effects over the life of the mission caused by radiation or other sources have been minimal, with only one of the 18 measures displaying a convincing deterioration trend. Ultimately, we demonstrate that DAVIS 240C Event-based Vision Sensors have a high aptitude for surviving long-term space flight.

## 1. Introduction

Event-based Vision Sensors (EVS) [1], or neuromorphic vision sensors, are a fundamentally different type of camera than traditional frame-based cameras that show significant potential in space-based applications. EVS cameras are inspired by biological retinas. Each pixel in the camera reports data asynchronously. Each pixel nominally reports data only if the scene observed by the pixel experiences a change in luminosity. This asynchronous and change-driven data collection differs from traditional frame-based cameras by offering a wide dynamic range and high temporal resolution while maintaining low power requirements and a comparatively low data rate. These factors combine to make EVS cameras a potent alternative to traditional frame-based cameras in the space domain.

Falcon Neuro is an experimental payload designed to investigate the capabilities of EVS cameras in the space domain when applied to Earth, atmospheric, and space observations from orbit [2]. Mounted on the International Space Station Columbus Module, Falcon Neuro has been active for approximately three years, operating two DAVIS 240C EVS cameras [3].

The purpose of this study is to evaluate the durability of the Falcon Neuro EVS cameras by measuring the degradation of the Falcon Neuro payload system over a three-year mission. Falcon Neuro was permanently decommissioned in August of 2025.

### 1.1. Related Work

While the high potential of EVS cameras in space-based applications and for space situational awareness has been recognized [2,4,5], there has never been a long-term study analyzing the survivability of an EVS camera in an uncontrolled, space-based environment. Radiation has been applied to EVS cameras of multiple models in order to test for radiation tolerance [6]. However, this study applied extreme doses of radiation over short time periods. While this offers insight into the resilience of EVS cameras, such a short-period, high-intensity test is not reflective of the uncontrolled environment aboard the ISS over a long-term mission.

Noise reduction is a vital part of EVS analysis, and optimizing signal-to-noise ratios is a recurrent theme in the EVS literature [7,8,9]. However, the analysis techniques presented in this paper are aimed at isolating noise and using it as a diagnostic tool to reveal long-term trends rather than simply removing it. Errors and noise in EVS sensors are distinct and not analogous to traditional frame-based cameras [7], and so different approaches must be utilized.

Finally, while there has been significant work in the field of hardening silicon-based integrated circuits and memory devices against radiation in order to make them more suitable to the space environment [10,11,12,13], the DAVIS 240C does not utilize any hardening techniques in its architecture or construction and only has limited physical shielding protecting it from radiation. In addition, the DAVIS 240C has a significantly different architecture and design than traditional CCD or CMOS cameras, meaning its reaction to radiation cannot be accurately estimated by observing CCD or CMOS radiation degradation.

### 1.2. Background

**Figure 1:**
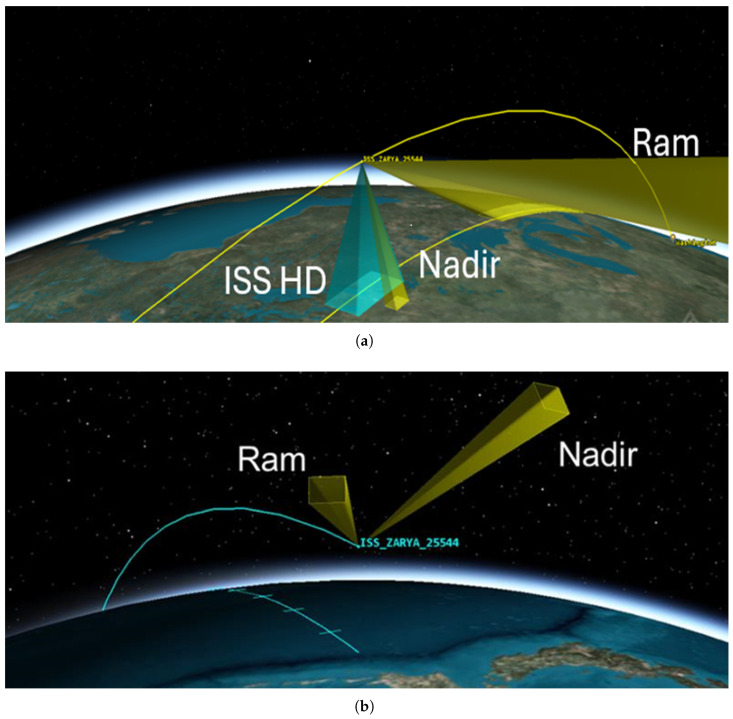
Field of view of the Falcon Neuro Ram and Nadir cameras. (**a**) shows the fields of view for the Ram and Nadir cameras in the original mounting position (2022–2023), with the Nadir camera pointing earthward and the Ram camera pointed in the bow direction of the ISS. In addition, the ISS HD camera is highlighted to show the Nadir camera’s comparative field of view and provide context. The yellow line represents the ISS flight path, with the second yellow line on the ground representing the directly nadir ground path of the ISS. (**b**) shows the fields of view for the Ram and Nadir cameras in the new position (2024–present). Both Ram and Nadir cameras are observing only the space environment with no views of Earth or Earth’s atmosphere. Nadir is pointing in the starboard direction and canted to zenith, and Ram is still pointed in approximately the bow direction. In the new position, neither camera is capable of seeing any part of the Earth. The blue line represents the ISS flight path.

The Falcon Neuro system is a manually commanded system containing two DAVIS 240C cameras, positioned roughly orthogonal to one another. The DAVIS 240C camera utilized in Falcon Neuro is a first-generation Event Vision Sensor developed by iniVation [14]. The DAVIS 240C is a 240 × 180 pixel EVS camera utilizing an arbiter. Each pixel uses a mixture of analog and digital circuitry to record changes in brightness independent of neighboring pixels. Each change is read one at a time by the arbiter, which sends the recorded event off the chip. All events are monotonically increasing in time. Falcon Neuro was launched in December 2021 and saw first light on 2 January 2022. Since then, Neuro collected data at semi-regular intervals until being moved to a new location aboard the ISS on 25 August 2023. On 30 May 2024, the Falcon Neuro pallet was powered back up, and Falcon Neuro began manually taking data once again at semi-regular intervals in its new location. The intermittent nature of data collection is a direct result of operator availability. Over the course of the mission, Falcon Neuro has recorded 979 min of data in total. However, given the experimental nature of the camera, internal hardware biases were frequently adjusted in order to find the subjective ‘most satisfactory’ bias settings. This bias setting allowed for the highest volume of data to be collected from orbit while minimizing background noise and reliably detecting known signal sources, such as cities or bright stars. This became the most common bias setting, referred to as Standard Biases. In total, 544 min of data were collected using Standard Biases. In our analysis, we will only consider the Standard Biases, as including all bias settings introduces additional variables that are not attributable to degradation effects. The two cameras are named Ram and Nadir, deriving their names from their respective fields of view in the initial position seen in Figure 1. When Falcon Neuro was moved in 2023, the Ram and Nadir cameras had their fields of view changed. The Ram camera continued to look in the approximate direction of travel of the ISS, although the elevation angle increased such that Ram was no longer capable of seeing the limb of the Earth, instead looking exclusively out into space. Similarly, the Nadir camera was shifted such that it could no longer see the Earth, and is instead looking out into space in the starboard and zenith direction.

**Figure 2:**
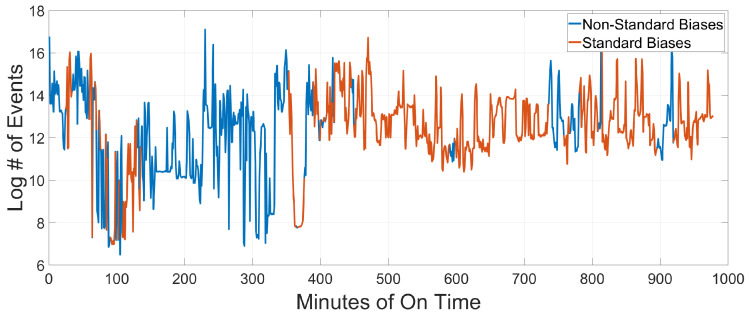
Log # of Events vs Total Minutes of On Time for Standard vs Non-Standard Biases across the life of the mission in both the Ram and the Nadir cameras. Standard Biases are highlighted in orange, while Non-Standard Biases are blue. While there are significant holes in the continuity of data collected with Standard Biases, the vast majority of data was collected on Standard Biases for both cameras.

In analyzing the data collected by Falcon Neuro across multiple scenes, it is important to note that EVS cameras can experience high levels of noise, particularly in low-light environments—although adjustment of biases can reduce this effect by reducing overall camera sensitivity, along with other biasing techniques [8]. This effect is of particular consideration in the new position, where both Ram and Nadir are looking out into space—an inherently low-light scene. The noise sources include the discrete nature of photons and electrons, transistor mismatch, changes to the circuitry across multiple biases, or dark noise. The result of this is that the noise profile of EVS cameras is extremely difficult to model—particularly when comparing different bias settings [5,8,15]. In particular, biases can have a large effect on the quantity of noise [8]. By normalizing for biases by only considering datasets that utilized Standard Biases, we can eliminate the noise quantity fluctuations that appear as a result of different biases. The total minutes recorded with Standard Biases can be seen in Figure 2. However, the inherent high level of noise—even with Standard Biases—provides an excellent opportunity for long-term analysis of the noise itself. EVS noise often appears with common or identifiable features that make noise effects entirely distinct from signal events. By isolating these specific noise behaviors, we are able to reliably isolate noise without confusing it with signal. As a result of the distinct noise behaviors and the difficulty in modeling EVS cameras, we will take a ‘top-down’ empirical approach to analyzing degradation in the Falcon Neuro camera, analyzing the data to produce conclusions about the camera as a whole. This type of top-down approach has shown promising results in signal extraction [9,16]. However, in this investigation, we are not interested in identifying signal. Instead, we are using the top-down approach to reliably isolate noise with known characteristics. Additionally, the purpose of this work is not to produce a model for EVS cameras but rather to take a directed empirical approach to define lifetime change in the Falcon Neuro system, using the unique noise features generated by the Falcon Neuro DAVIS 240C EVS cameras exclusively.

## 2. Methods

**Figure A1:**
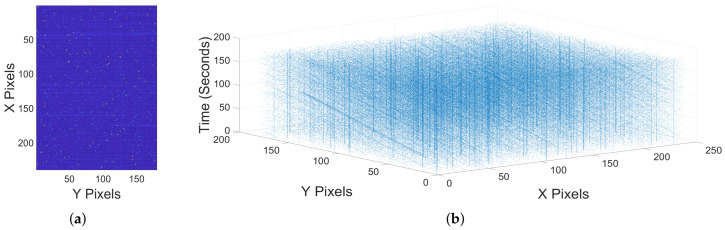
(**a**) Time-integrated two-dimensional frame of raw EVS data. Pixels range from blue to yellow, with blue representing pixels that recorded relatively few events and yellow representing pixels that recorded relatively many events. (**b**) Three-dimensional figure that represents the same data as the 2D image, while keeping time information related to each event intact. The 3D image is organized with X pixels and Y pixels on the x- and y-axes and time information on the z-axis. Persistent features and details are vastly more visible in the 3D image.

Event-based Vision Sensors detect illuminance changes in the scene. They do so at the pixel level, with a mix of dedicated analog and digital circuitry. Such circuits provide advanced signal processing functions with high power efficiency but they have complex noise signatures. As a consequence, change detections—or events—reported by the camera are often caused by noise in the pixel circuit rather than actual illuminance changes. Events constituting a real signal are created when a pixel observes a change in light intensity. This is a marked difference from traditional frame-based sensors, which measure how many photons were collected on each pixel in a given time period. Over the course of the methodology section, we will use three different example datasets, all included in the supplemental data: the Ram Stars Set, the Lightning Set, and the RoCo Set. The first and most common example dataset is the Ram Stars Set, shown in its entirety in Figure A1. The other two datasets are used to create clear examples in the RoCo section below.

### 2.1. Noise or Signal?

We propose that there are two data-driven methods through which to measure EVS degradation: signal or noise. In the case of signal, if the number of signal events generated while looking at the same signal source decreases, then the camera has degraded. Similarly, if the noise profile produces significantly different levels of noise over the life of the camera, then the camera has degraded. Notably, noise does not rely on observing the same real events like signal does and can be used as a comparative measuring stick regardless of real features in the camera field of view (FOV). Due to the intermittent schedule of data collection and the move to the new position midway through the mission, Falcon Neuro has observed a wide variety of scenes over its lifetime so far, with very few signal sources being repeated consistently in either Ram or Nadir since the start of the mission in 2022. In addition, signal is extremely difficult to define with high certainty in EVS cameras, particularly while observing relatively dim objects like stars [5]. Because no detailed analysis has been done on the dynamic range of the Falcon Neuro system, it is extremely difficult to reliably determine what constitutes signal. When timing precision is added to the equation, the challenge of isolating signal becomes even greater. Typically, Falcon Neuro has a timing accuracy of no better than 1 s between the actual signal change producing an event and the recorded timestamp [17]. By contrast, the noise profiles of both the Ram and Nadir cameras with Standard Biases consistently produce noise with definable characteristics, regardless of other factors. These definable noise characteristics make it possible to reliably isolate noise behaviors and measure how they change over the life of the camera. As such, we have chosen noise as the practical metric to assess degradation in the Falcon Neuro system.

Across all data in the Falcon Neuro dataset, four types of uniquely characteristic noise were consistently observed. The four types of noise are as follows:

- Hot Pixels—pixels that record more events than expected;
- Boring Pixels—pixels that record events at a constant rate;
- Row/Column (RoCo) Errors—entire rows or columns of pixels that record events in an unusually short time period;
- Lonely Events—events that occur in a highly isolated manner in time or space.

In addition to these four noise types, we have also chosen to isolate Cold Pixels—pixels that record fewer events than expected. In the case of Cold Pixels, we are not necessarily isolating noise. In fact, Cold Pixels tend to be less sensitive to noise—including random background noise—than the four noise types mentioned. However, by detecting Cold Pixels and observing their event rates over the Falcon Neuro mission, we can see if pixels have become significantly less sensitive or have died completely, providing a useful measure for payload degradation. Even though Cold Pixels do not necessarily represent true noise, we will still refer to Cold Pixels as a noise type moving forward in our analysis.

In the context of this paper, a false positive is a true signal event that has been confused for noise, and a false negative is a noise event that has not been isolated. Due to the extreme difficulty already discussed in identifying a true signal at the detection limits, false positives and false negatives are often identified visually using context and experience. In order to ensure that as little signal as possible is confused for noise, each algorithm uses subjectively ‘gentle’ parameters, defined as parameters that tend towards producing false negatives rather than false positives. The parameters used are determined using a variety of methods discussed in Appendix A.1 to produce consistently small quantities of false positives across the full Falcon Neuro data collection, regardless of scene.

### 2.2. Algorithms

**Figure 3:**
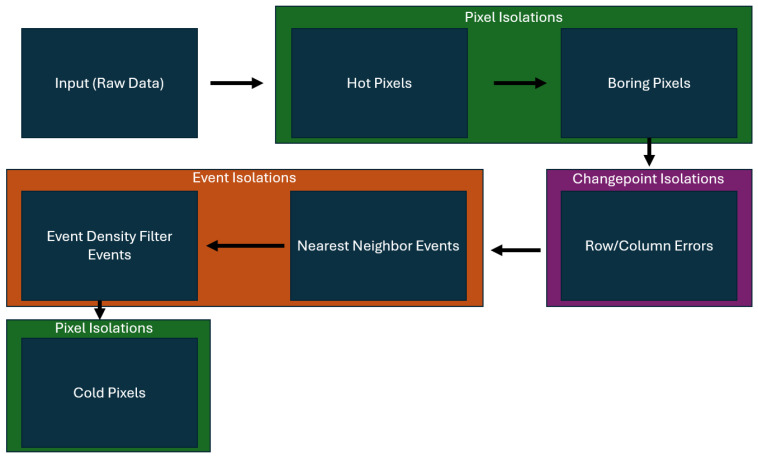
Pipeline showing the order of algorithms used to isolate individual noise types.

The algorithms used in this paper are applied to the raw Falcon Neuro data sequentially, as seen in Figure 3. As the input data is passed through each filter, the various noise types are isolated and removed from the input data for further analysis. By isolating and removing noise types as we progress through the pipeline, each filter utilized improves the runtime and accuracy of the next downstream filter. Cold Pixels are calculated last, once all other noise types have been isolated out of the input data. For the other noise types, it is important to note that the nearest neighbor (NNb) filter and the Event Density Filter (EDF) both target Lonely Events. All other filters share the same name as the noise type they are meant to isolate.

In the noise isolation pipeline, there are three types of noise isolation. The first type is a pixel isolation, where entire pixels that exhibit hot or boring behavior are isolated and removed from the data stream. The next type of noise isolation is a changepoint isolation, in which a row or column of data is removed due to a prevalence of row/column (RoCo) errors using a changepoint algorithm. Finally, the last noise isolation method is an event isolation, where individual events are identified as lonely and isolated for further study. The identification of lonely events is broken into two separate isolation algorithms—the nearest neighbor (NNb) algorithm and the Event Density Filter (EDF) algorithm.

The Falcon Neuro system typically took data in 30–180 s durations, with 180 s being the most common. For our analysis, the Falcon Neuro dataset under Standard Biases is broken up into sixty-second segments, and for each segment, each noise type is isolated and separated from the input data. For each sixty-second segment, the number of events isolated is calculated for each noise type. These values can then be observed and compared over the complete life of Falcon Neuro.

In the following sections, each noise algorithm is described in greater detail. For simplicity, each pixel address will be denoted as: $\left\{T_{ij}|1\leq i\leq 240,1\leq j\leq 180\right\}$ where *T* represents the Target Pixel.

#### 2.2.1. Hot Pixels

Hot Pixels are the first type of noise isolated, as seen in Figure 3. To isolate Hot Pixels, the total number of events in each individual pixel is summed for each sixty-second segment of data. Then, the average number of events and the standard deviation of events in the eight “nearest neighbor” (i.e., adjacent) pixels are calculated. Then, if the target pixel produces *n* standard deviations more events than the average of its nearest neighbor pixels, the target pixel is isolated from the Hot Pixel algorithm input data. Let $\bar{N_{ij}}$ be the average number of events in the nearest neighbor pixels, and let σ be the standard deviation in the events of the nearest neighbor pixels.

(1) $$A\;\mathrm{pixel}\;\mathrm{is}\;\mathrm{hot}\;\mathrm{if}\;T_{ij}>\bar{N}+5.5\sigma$$

**Figure A2:**
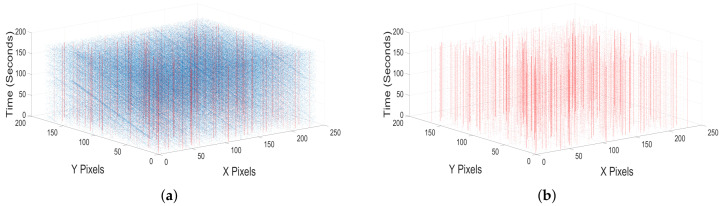
(**a**) A 3D image with the input data to the Hot Pixel algorithm in blue and the identified Hot Pixels in red. (**b**) A 3D image of the Hot Pixels isolated from the raw data.

We have chosen to use a parameter of 5.5. Details on parameters can be found in Appendix A. The Hot Pixel algorithm is performed first in the noise isolation pipeline because it is computationally simple and not prone to false positives, giving a high confidence that only noise is being isolated. The results of the Hot Pixel algorithm isolation can also be seen in Figure A2 in Appendix A.

#### 2.2.2. Boring Pixels

Boring pixels are the second type of noise isolated through the pipeline shown in Figure 3. Boring pixels are pixels where the time between each recorded event is unusually consistent, regardless of changes in the scene. Boring pixels are identified using a two-step process. The first step is candidature and the second step is confirmation.

In the candidature step, each pixel in the focal plane is observed individually. For a given target pixel, $T_{ij}$, the time of each recorded event in $T_{ij}$ is observed. Then, the time between each event and the closest preceding event in time is calculated. Let *P* be the set of all event times in a single target pixel, $T_{ij}$, with monotonically increasing time such that $P_{k}>P_{k-1}$. Finally, let ISI = Inter-Spike Interval, where ISI represents the time between events in a single pixel. Then, the time between events is the set found using Equation (2).

(2) $$\left\{ISI\right\}=\left\{P_{k}-P_{k-1}\right\}$$

In practice, we feed 60 s segments of data into each algorithm. Therefore, 0≤k≤60. Due to the initial event in any given target pixel having no comparison point, the cumulative length of the set {ISI} is t−1. A single ISI set is created for each individual pixel. Then, to finish the candidature step, the standard deviation σ is calculated for each ISI set. If σ is sufficiently low, then the target pixel is flagged as a boring pixel candidate. This threshold is arbitrary, but in keeping with the three primary purposes of parameter selection, the standard deviation threshold is set to 1. This value was found experimentally, and succeeds in consistently isolating boring pixels while limiting false positives.

(3) A target pixel is a boring candidate iff σ(ISI)<1

**Figure A3:**
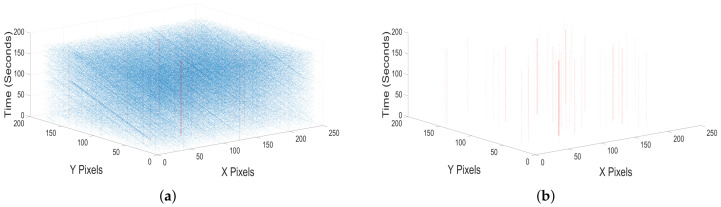
(**a**) A 3D image with the input data to the Boring Pixel algorithm in blue and the identified Boring Pixels in red. (**b**) A 3D image of the Boring Pixels isolated from the raw data.

Each candidate boring pixel is given a flag value of either 1 or 0, depending on if the pixel is a candidate or not. It is entirely possible that a given pixel’s consistent ISI is caused by real scene effects. To prevent labeling these possible effects as noise when they are in fact signal elements, a second, confirmation process is included to identify boring pixels. The confirmation is a simple neighbor check based on how real events are observed. If more than 3 of the nearest neighbors of the target pixel $T_{ij}$ are also flagged as boring, then it is possible that the pixels are seeing a real signal change that is causing a consistent ISI. However, if fewer than 3 neighboring pixels are flagged as boring, then it is likely that the flagged boring pixel is actually boring, and is producing false events with a consistent ISI. In that case, the pixel is considered a Boring Pixel and is isolated from the data. The results of the Boring Pixel algorithm can be seen in Figure A3.

#### 2.2.3. Row/Column (RoCo) Errors

**Figure A5:**
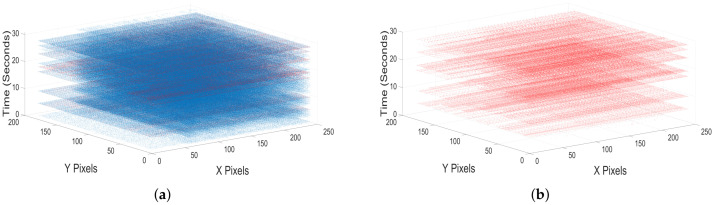
(**a**) A 3D image of RoCo Errors in the RoCo algorithm input data after the Hot Pixels and Boring Pixels have been isolated and removed in blue and the RoCo Errors in red. (**b**) A 3D image of the RoCo isolated from the data.

Hot and Boring Pixels are the only two noise types in the pipeline that are detected using pixel isolation. In comparison, RoCo errors are removed using a changepoint detection method [18]. RoCo errors appear as a single row or column recording an unusually high number of events in a very short time period. While the example we have chosen to show in this section has a multitude of RoCo errors occurring in a short time window across different rows and columns, RoCo errors can also happen in isolation, with only a single row or column in a given time window producing a RoCo error and all other rows and columns behaving normally. To detect RoCo errors, two sets are created, {R} and {C}, standing for row and column, respectively. The row set, {R}, is associated with the x-coordinates in the camera and contains 240 available slots. Similarly, the column set is associated with the y-coordinates in the camera, and contains 180 slots. For each event, the x and y-coordinates are extracted, *i* and *j*, respectively. Then, ${\left\{R\right\}}_{i}={\left\{R\right\}}_{i}+1$ and $\left\{C_{j}\right\}=\left\{C_{j}\right\}+1$. This is done as a continuous summation for each event, without first resetting the {R} and {C} sets. Additionally, for each event, both the row and column sets have a constant value *D* subtracted from each index in the vector. This value is chosen based on the evidence in Figure A9 in Appendix A, and is set at 0.5. This applies a linear decay to the row and column sets. However, no value in {R} or {C} is allowed to decay below 0. If a particular row or column set index crosses a threshold, *H*, then an unusually high number of events has occurred in that row or column without decay caused by events in other rows or columns. Once a particular index in the row or column set has crossed the threshold value, *H*, we know that a RoCo error has started and is ongoing. *H* is chosen based on the evidence in Figure A9 and is set to 10. As such, every subsequent event in an index with a sum above *H* is flagged and isolated as a RoCo event until the linear decay forces the heightened row or column set index sum back below the threshold value. Let $x_{ij}$ be an event with 0≤i≤240 and 0≤j≤180. Now, the equation for the RoCo algorithm in the Row set looks like the following:

(4) $$\mathrm{For}\;\mathrm{each}\;\mathrm{event}\;x_{ij},{\left\{R\right\}}_{i}={\left\{R\right\}}_{i}+1$$

(5) $$\mathrm{For}\;\mathrm{each}\;\mathrm{event}\;x_{ij}>0,\left\{R\right\}=\left\{R\right\}-D$$

(6) $$\mathrm{For}\;x_{ij},x_{ij}\;\mathrm{is}\;a\;\mathrm{RoCo}\;\mathrm{Error}\;\mathrm{iff}\;{\left\{R\right\}}_{i}>H$$

where *D* is the linear decay, and *H* is the changepoint threshold, and each event occurs earlier in time than the next following event. This equation is mirrored identically for the column set. We have chosen to use a linear decay rather than an exponential decay because it allows RoCo errors to remain above the threshold value until the RoCo error has occurred in its entirety with minimal need to fine-tune parameters. This effect can be seen in Figure A9. The effect of the RoCo Error algorithm can be seen in Figure A5.

#### 2.2.4. NNb Events

The next pipeline algorithm is an event isolation algorithm—the nearest neighbor event algorithm (NNb) [7]. Unlike the previous three algorithms, which relied on changepoint-based or pixel-based isolation techniques, we are now observing and evaluating each individual event in the data stream to determine if it is noise or not.

NNb Events are calculated on a pass/fail basis, where every event is tested by the filter and sorted into a ‘NNb Noise Event’ matrix and a ‘Not NNb Noise Event’ matrix. First, the Average Inter-Spike Interval (AISI) is calculated by dividing the total time by the total number of events, AISI=Total Time/# of events. This value is given in seconds per event. Then, each event is fed into the algorithm sequentially in the format $E_{i}=\left\{x_{i},y_{i},t_{i}\right\}$, where *i* is the event index and $t_{i}$ is monotonically increasing as *i* increases. The range of *i* varies widely depending on how many events were recorded in a data segment. However, 1≤i≤ Final Event Index. For each event $E_{i}$, the most recent single time prior to $t_{i}$ is extracted from the eight nearest neighbor pixels collected into the set N={Most Recent Neighboring Pixel Times}. Now, we subtract the Timing Threshold value from $t_{i}$, where Timing Threshold=c∗AISI, with *c* being a parameter discussed in greater detail in Appendix A. NNb noise events are most easily confused with especially dim signal events, like stars. As such, the NNb Timing Threshold parameter is set in order to minimize false positives when observing known dim signal events. Then, if at least five of the nearest eight neighbors have a most recent time value lesser than $t_{i}$ − Timing Threshold, it is likely that the observed event is an isolated event and did not occur as a part of a true signal. This is done in accordance with the following equation:

(7) $$E_{i}\;\mathrm{is}\;a\;\mathrm{noise}\;\mathrm{event}\;\mathrm{iff}:\;\left\{\#\mathrm{of}\;\mathrm{events}\in N|N\leq t_{i}-\mathrm{Timing}\;\mathrm{Threshold}\right\}<3$$

**Figure A6:**
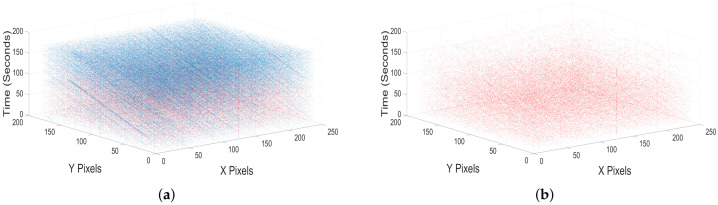
(**a**) A 3D image of NNb noise events with the original data after the Hot Pixels, Boring Pixels, and RoCo Errors have been isolated and removed in blue and the NNb Noise Events in red. (**b**) A 3D image of the NNb Noise Events isolated from the data.

In this way, we are able to isolate much of the background noise. The results of the NNb algorithm are seen in Figure A6. Due to the relatively small neighborhood being used for the NNb algorithm, and the relatively wide time window, the NNb algorithm is best suited for separating pixels that are extremely isolated in time or space while remaining computationally efficient.

#### 2.2.5. EDF Events

The Event Density Function (EDF) Events algorithm is a modification of the NNb algorithm discussed in the previous section, and is fundamentally similar in purpose. However, unlike the NNb algorithm, the EDF algorithm allows for an adjustable radius and an adjustable time window. For each event, a ‘box’ with dimensions *x*, *y*, and *t* is drawn. Then, the density of events within the target box is calculated and compared to the overall density of the complete dataset being observed.

**Figure A7:**
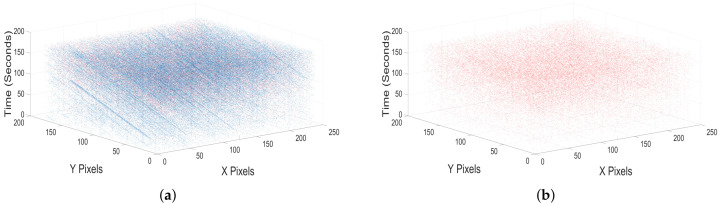
(**a**) A 3D image of EDF noise events with the original data after the Hot Pixels, Boring Pixels, RoCo Errors, and NNb Noise Events have been isolated and removed in blue and the EDF Noise Events in red. (**b**) A 3D image of the EDF Noise Events isolated from the data.

**Figure A9:**
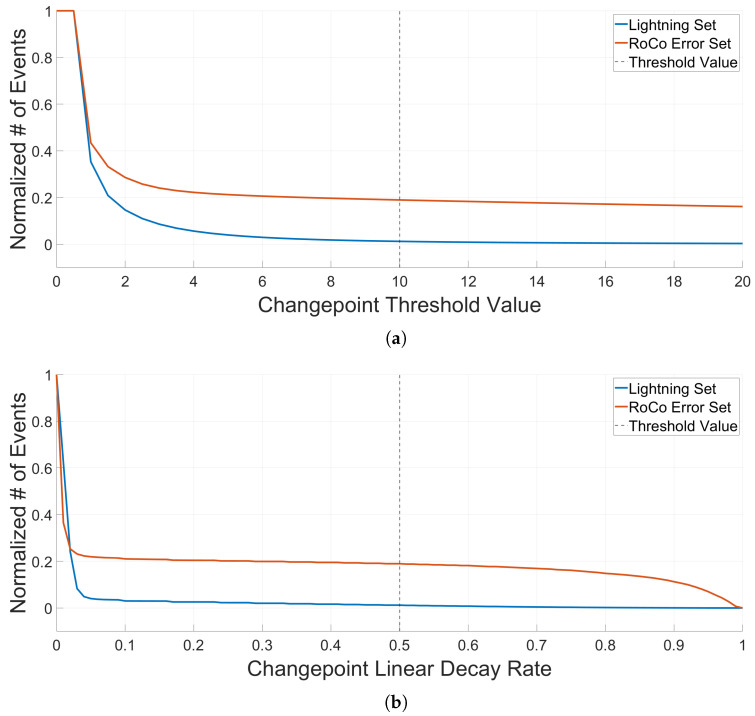
RoCo plots showing two datasets. In orange is the RoCo sample dataset, which contains high volumes of RoCo errors. In blue is the lightning sample dataset. These two sets are compared because lightning is the most common type of data that can be confused as a RoCo error. The normalized number of events isolated as a RoCo error is on the y-axis. (**a**) The x-axis shows the changepoint critical threshold value. The plot shows that, after a threshold value of 1, the number of events isolated tends to level out considerably. We have chosen a threshold value of 10. (**b**) The x-axis shows the changepoint linear decay rate. The plot shows that, after a decay rate of 0.05, the number of events isolated tends to level out considerably. Any decay rate greater than approximately 0.8 shows a much lower number of events being isolated as RoCo errors. We have chosen to use a decay rate of 0.5.

**Figure A11:**
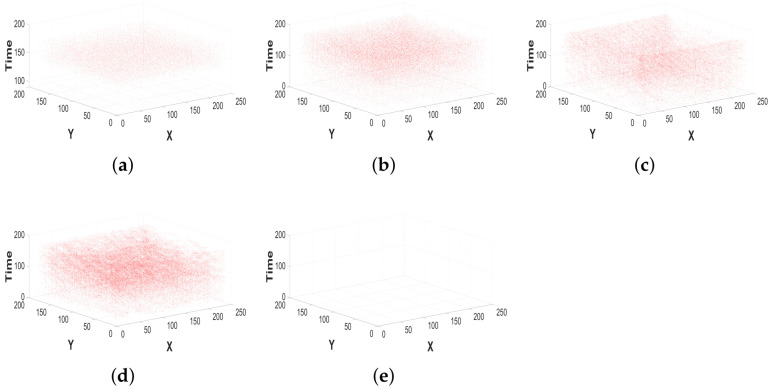
Three-dimensional plots showing the isolated EDF Noise Events as the time window and radius parameters are changed. (**a**) Time parameter = 1, Radius parameter = 2. (**b**) Time parameter = 1, Radius parameter = 5. (**c**) Time parameter = 1, Radius parameter = 15. (**d**) Time parameter = 10, Radius parameter = 5. (**e**) Time parameter = 0.1, Radius parameter = 5. Time is in seconds. The parameters that produced the highest number of noise events isolated with the fewest false positives were those used in (**b**).

EDF Events are calculated on a pass/fail basis, where every event is tested by the filter and sorted into an ‘EDF Noise Event’ matrix and a ‘Not EDF Noise Event’ matrix. First, a normalized density is calculated on the input data Density=#of Events/(Total Time∗240∗180). Then, each event is fed into the EDF algorithm sequentially in the format $E_{i}=\left\{x_{i},y_{i},t_{i}\right\}$, where *i* is the event index and $t_{i}$ is monotonically increasing as *i* increases. For each event, a box of radius 5 pixels and a time window of 1 s is drawn around the event. Because the events are fed into the algorithm sequentially in time, the time window around the focus event only takes past events into account. Changing the parameters has a somewhat mild difference on the remaining data—which can be further explored in Figure A11 in Appendix A—so parameters were chosen to maximize EDF noise events without creating high quantities of visually clear false positives. For each event, if the local normalized density surrounding the target event is less than 75% of the total normalized data density, then the event is considered an EDF noise event and is isolated. In general, the ratio of local normalized event density around real events compared to the total normalized event density will be greater than 1, so the 75% value is only in place to prevent false positives in the less common case when the ratio of local normalized density and the total normalized density is close to 1. The results of the EDF filter are seen in Figure A7 in Appendix A.

#### 2.2.6. Cold Pixels

**Figure A12:**
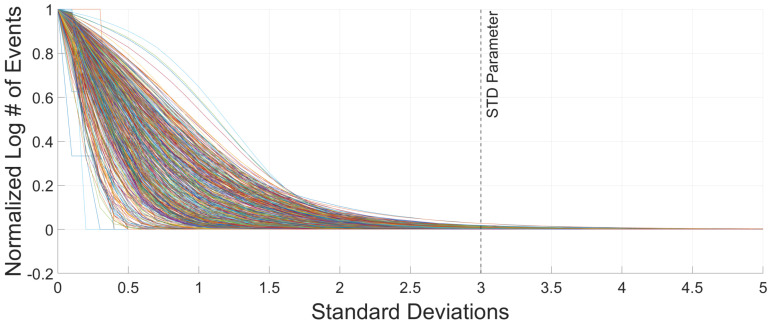
Cold Pixel plot demonstrating how the normalized number of events considered ‘cold’ varies as the standard deviation parameter is changed across all datasets using Standard Biases. Each line represents a single 60 s dataset. The y-axis represents the normalized number of events (not pixels) that have been considered cold events. Each cold event ultimately belongs to a Cold Pixel. The x-axis represents the standard deviations as used in Equation (1). As the standard deviation increases, the number of cold events decreases. Ultimately, the standard deviation of Equation (1) was set to three, because minor adjustments to the standard deviation parameter produce insignificant changes in the results. Additionally, choosing a standard deviation of three isolates as many Cold Pixel events as possible, while still allowing for minor changes in the parameter and minimizing false positives.

Cold Pixels encompass the group of pixels that are under-responsive or completely unresponsive to signal and noise. This manifests as an unusually low event rate in these particular pixels relative to neighboring pixels. Therefore, Cold Pixels are found by comparison to neighboring pixels. Cold Pixels are also calculated last, after all of the other noise types have been isolated out of the original data. This prevents significant outliers, such as events from Hot Pixels or Boring Pixels from producing inaccurate results. In general, Cold Pixels tend to be isolated from one another, meaning that neighboring pixels are not cold. The detection method for Cold Pixels is a straightforward neighbor comparison as shown below, where we define the following:

$$\begin{array}{c} T_{ij}=\mathrm{Target}\;\mathrm{Pixel}\;\#\;\mathrm{of}\;\mathrm{events},\\ \bar{N}=\mathrm{Mean}\;\#\;\mathrm{of}\;\mathrm{events},\mathrm{and}\\ \sigma =\mathrm{Standard}\;\mathrm{Deviation}\;\mathrm{of}\;\mathrm{Nearest}\;\mathrm{Neighbor}\;\mathrm{Pixels}\;\#\;\mathrm{of}\;\mathrm{events} \end{array}$$

(8) $$A\;\mathrm{pixel}\;\mathrm{is}\;\mathrm{cold}\;\mathrm{iff}\;T_{ij}<\bar{N}-3\sigma$$

where 0≤i≤240 and 0≤j≤180. This equation simply measures whether each pixel is producing significantly fewer events than the nearest surrounding pixels. When the Hot and Boring Pixel algorithms operate on the data, they remove all events in isolated pixels, creating pixels that look cold or dead when observed in isolation. In order to resolve this, each pixel isolated by the Boring and Hot algorithms is temporarily assigned events equal to the average number of events in the Cold Pixel algorithm input data. This prevents the Cold Pixel algorithm from accidentally labeling pixels already isolated as Hot or Boring as Cold. The standard deviation parameter is selected to be 3 based on the evidence in Figure A12. By setting the standard deviation coefficient to 3, we can ensure that small variations in the standard deviation will not produce significant changes in our results.

## 3. Results

Now we return to the primary purpose of the paper: evaluating Falcon Neuro degradation over the lifespan of the camera. In order to do this, we will ingest all of the raw data that was collected with Standard Biases sequentially in the order the data was collected. Then, we will divide the raw data into 60 s segments. Each 60 s segment will be measured for Cold Pixels first, and then each 60 s segment will be pushed through the noise isolation pipeline of Figure 3. After the segment has had each noise type isolated out through the pipeline, we will measure the number of Cold Pixels in the remaining data. Each 60 s segment will produce a number of events of each noise type, producing six values for each camera in addition to the number of events recorded in the original data. Additionally, for Hot Pixels, Boring Pixels, and Cold Pixels, a count of the number of pixels classified as each type will be collected, providing three values for each camera. This produces a total of 18 categories by which to measure lifetime degradation. Then, we will search these 18 categories for statistically significant monotonic trends, which would be indicative of real changes to the noise levels over the life of the camera. We will measure for monotonic trends using a Mann–Kendall (MK) test for monotonic trends at the 95% confidence level. We are using the method described in [19] to conduct the MK test. For both the Ram and Nadir cameras under Standard Biases, raw data are divided into two main categories: all lifetime data and 2024 data. While all lifetime data saw a variety of FOVs focused on both Earth and space, the data taken in 2024 only observed an FOV focused on space. By separating the data in this way, we are able to observe the approximately normal scene observed by the 2024 data rather than the chaotic and varied scenes observed by the lifetime data. In addition, we will consider correlation coefficients between the number of events in the input data and the number of events isolated by each algorithm. This will help demonstrate to what extent each noise type is affected by the overall event rate of the camera. Finally, during the construction of Falcon Neuro, an Engineering Design Unit (EDU) was produced as a clone of Falcon Neuro. The EDU has remained in storage for the duration of the Falcon Neuro mission, and has not suffered degradation due to the space environment. In Appendix B, we have provided comparison plots showing noise levels in the EDU and in Falcon Neuro. The EDU comparison plots display an extremely high level of similarity between the EDU and Falcon Neuro, suggesting Falcon Neuro has not degraded.

### 3.1. Ram

#### 3.1.1. Ram Default Biases—Full Mission

**Figure 4:**
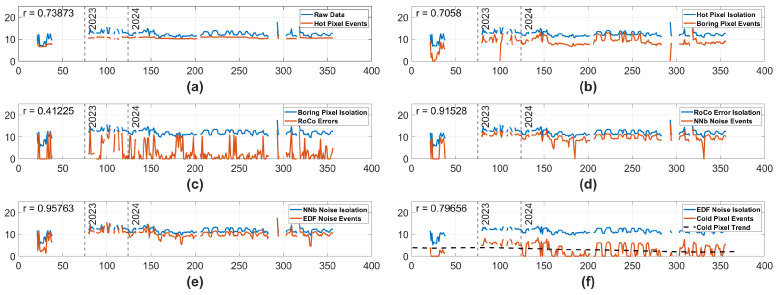
Data from the Ram camera using Standard Biases over the complete life of the mission. The y-axis represents the natural log # of events, and the x-axis represents minutes of on time. The blue lines represent the log number of input events, and the orange lines represent the log # of isolated events. Each blue line representing input events is the raw data with all previous pipeline noise types isolated away. In the case of (**f**), the black dashed line displays a statistically significant trend in the isolated pixels. Vertical dashed lines are labeled 2023 and 2024, indicating which year the data was taken in. In the top left corner of each subplot is an r number, representing the correlation between the input data and the isolated data. (**a**) Hot Pixel isolation. (**b**) Boring Pixel isolation. (**c**) RoCo Error isolation. (**d**) NNb Noise Events isolation. (**e**) EDF Noise Events isolation. (**f**) Cold Pixel isolation with the blue line representing the input data with all other noise types isolated out of the original data.

When observing the results seen in Figure 4, we can see that all noise isolation algorithms have a high correlation to the input data associated with each isolation type, with the exception of RoCo errors. Given the reality that the observable scene changed so dramatically over the course of the mission, as seen in Figure 1, a high correlation between the noise events and the input data events may cause unreliable trends. It is possible that the trend in the Cold Pixels seen in Figure 4 is caused by correlation and high variance rather than by real degradation. In contrast, the RoCo Errors in the Ram camera have a relatively low correlation to the input data, and the RoCo errors demonstrate no statistically significant trend, strongly indicating that there is no degradation in the RoCo Errors over the full mission.

#### 3.1.2. Ram 2024 Default Biases

**Figure 5:**
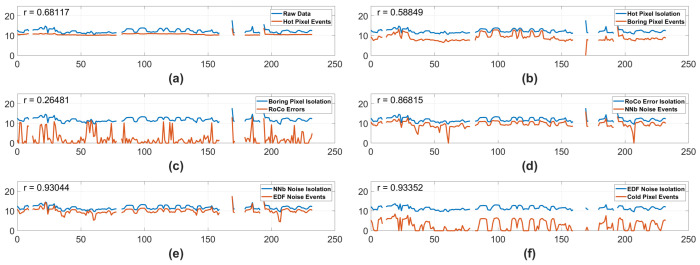
Data from the Ram camera using Standard Biases in 2024. The y-axis represents the log # of events and the x-axis represents minutes of on time. The blue lines represent the log # of input events and the orange lines represent the log number of isolated events. Each blue line representing input events is the raw data with all previous pipeline noise types isolated away. In the top left corner of each subplot is an r number, representing the correlation between the input data and the isolated data. (**a**) Hot Pixel isolation. (**b**) Boring Pixel isolation. (**c**) RoCo Error isolation. (**d**) NNb Noise Events isolation. (**e**) EDF Noise Events isolation. (**f**) Cold Pixel isolation with the blue representing the input data, with all other noise types isolated out of the original data.

Now, as seen in Figure 5, we will only take the data collected by the Ram camera with Standard Biases during 2024. This approximately normalizes the scene observed in each separate minute of data by only considering data taken while observing a space-focused FOV. Without the influence of the scene impacting potential trends, we will again perform an MK test. With this data, we are able to confirm that none of the six noise types experienced a monotonic trend either up or down. In fact, none of the six noise types are significantly different in any way over the course of 2024, according to the MK test. This, in addition to the lack of a trend seen in the RoCo data across all the full time span regardless of scene, implies that the Ram camera has not degraded across any of our six measuring methods.

#### 3.1.3. Ram Pixel Counts

**Figure 6:**
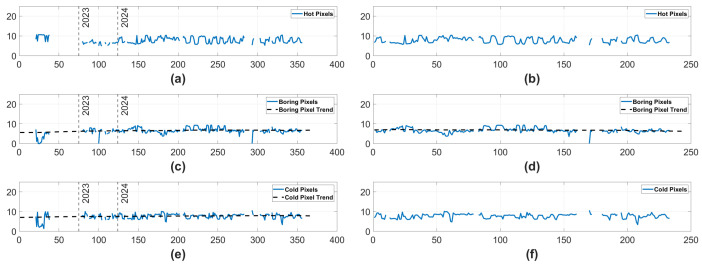
The log number of pixels isolated in the Hot, Boring, and Cold Pixel algorithms for the Ram camera. The y-axis represents the log # of pixels isolated, and the x-axis represents the minutes of on time. In (**a**,**c**,**e**), there are two vertical lines labeled 2023 and 2024, which represent which year the data was collected in. In the case of (**c**–**e**), a black dashed line displays a statistically significant trend in the data. The trend is monotonically increasing in (**c**,**e**), but is monotonically decreasing in (**d**). (**a**) Hot Pixels over the complete dataset. (**b**) Hot Pixels in 2024. (**c**) Boring Pixels over the complete dataset. (**d**) Boring Pixels in 2024. (**e**) Cold Pixels over the complete dataset. (**f**) Cold Pixels in 2024.

Next, we will observe the number of pixels isolated by the Hot, Boring, and Cold Pixel algorithms in each time step rather than events. These values are displayed in Figure 6. On the left hand side of the figure, Boring Pixels and Cold Pixels both display statistically significant monotonically increasing trends. In contrast, the right side of Figure 6 suggests that only the Boring Pixels displayed a statistically significant trend. Notably, the trend when only observing data from 2024 is actually a monotonically decreasing trend. While the Boring Pixels display a trend across all data and in the data from 2024, the opposite direction of the trends make it unclear how the camera has degraded, if it has actually degraded at all. In combination with the earlier results displayed in Figure 4 and Figure 5, and the fact that neither the Hot nor Cold Pixel counts carry a trend in 2024, it is likely that any degradation truly experienced by the Ram camera has been negligible.

### 3.2. Nadir

#### 3.2.1. Nadir Default Biases—Full Mission

**Figure 7:**
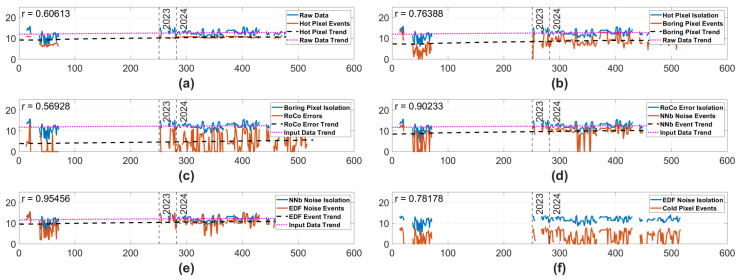
Data from the Nadir camera using Standard Biases over the complete life of the mission. The y-axis represents the log # of events, and the x-axis represents minutes of on time. The blue lines represent the log number of input events, and the orange lines represent the log # of isolated events. Each blue line representing input events is the raw data with all previous pipeline noise types isolated away. In the case of each tile except (**f**), the black dashed line displays a statistically significant trend in the isolated pixels, and the pink dashed line displays a statistically significant trend in the input data. Vertical dashed lines are labeled 2023 and 2024, indicating which year the data was taken in. In the top left corner of each subplot is an r number, representing the correlation between the input data and the isolated data. (**a**) Hot Pixel isolation. (**b**) Boring Pixel isolation. (**c**) RoCo Error isolation. (**d**) NNb Noise Events isolation. (**e**) EDF Noise Events isolation. (**f**) Cold Pixel isolation with the blue representing the input data, with all other noise types isolated out of the original data.

The results seen in Figure 7 are extremely similar to the Ram case, with generally high correlations and a comparatively low correlation in the RoCo Errors. Unlike the Ram case, there is a statistically significant trend in the Nadir RoCo Errors, suggesting some level of degradation is possible in the Nadir RoCo Errors. Also, unlike the Ram camera, the Nadir camera does demonstrate statistically significant trends in the Hot Pixel Events, Boring Pixel Events, NNb Noise Events, and EDF Noise Events. However, these trends exist in noise types that have high correlation with input data and may be affected by significant changes in scene over the life of the camera or even by the significant gap in Nadir data in 2022.

#### 3.2.2. Nadir 2024

**Figure 8:**
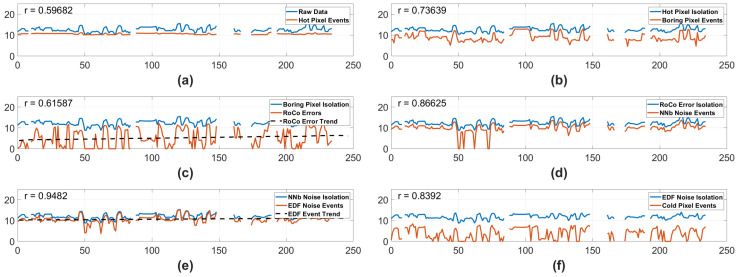
Data from the Nadir camera using Standard Biases in 2024. The y-axis represents the log # of events, and the x-axis represents minutes of on time. The blue lines represent the log # of input events, and the orange lines represent the log number of isolated events. Each blue line representing input events is the raw data with all previous pipeline noise types isolated away. In the case of (**c**,**e**), the black dashed line represents a statistically significant trend. In the top left corner of each subplot is an r number, representing the correlation between the input data and the isolated data. (**a**) Hot Pixel isolation. (**b**) Boring Pixel isolation. (**c**) RoCo Error isolation. (**d**) NNb Noise Events isolation. (**e**) EDF Noise Events isolation. (**f**) Cold Pixel isolation with the blue representing the input data with all other noise types isolated out of the input data.

When observing the Nadir data collected in 2024, many of the trends seen in the lifetime data dissapear. In this scenario, the RoCo Errors and EDF Noise Events exhibit statistically significant, monotonically increasing change. However, it is also important to note that the EDF Noise Events have the highest correlation to the input data. Even though the input data for the EDF noise events does not have a trend, it is possible that the high correlation accentuated otherwise minor effects from the variance, allowing a minor trend to appear in the EDF Noise Event data. In the case of the Nadir RoCo Errors, the correlation between input data and isolation data is much lower, and it is most likely that there truly was an increase in RoCo errors due to degradation, rather than any other effect. Given that only two of the tested noise types exhibited a statistically significant trend when normalizing for scene and bias in the Nadir camera, there is insufficient evidence to declare a complete deterioration of the camera. Additionally, the MK test in Figure 8 is conducted at the 95% confidence level. If the MK test is conducted again at the 99% confidence level rather than the 95% level, the trend in the EDF Noise Events disappear—meaning there may not be a significant monotonic trend in the EDF Noise Events at all. However, the statistically significant trend in the RoCo Errors remains even at the 99% confidence level. This further suggests that the EDF Noise Event trend may be caused by a high variance and a high correlation, rather than being caused by true degradation. Simultaneously, the 99% confidence MK test also shows that the RoCo Errors did experience a monotonic trend, strengthening the argument that the Nadir RoCo Errors did experience real degradation.

While it is possible that the camera degraded across the EDF Noise Events, it is also possible that the trend is affected by a high variance rather than degradation. In contrast, the RoCo Errors do not share a high correlation with the input data, and are therefore relatively decoupled from scene effects. Thus, the high-confidence trend in the Nadir RoCo Errors is most likely caused by some level of deterioration in the camera. Given the RoCo Error trend coupled with the other, less convincing trend of the EDF Noise Events, it is reasonable to conclude that the Nadir camera experienced a mild degradation in performance. This manifests primarily as a slightly higher number and frequency of RoCo error events—though this increase has not made a practical impact on the quality of data collected.

#### 3.2.3. Nadir Pixel Counts

**Figure 9:**
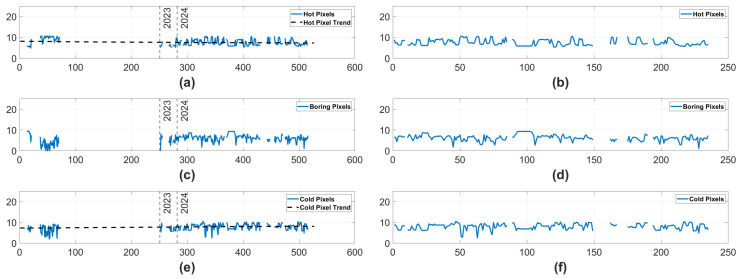
The log number of pixels isolated in the Hot, Boring, and Cold Pixel algorithms for the Nadir camera. The y-axis represents the log # of pixels isolated, and the x-axis represents the minutes of on time. In (**a**,**c**,**e**), there are two vertical lines labeled 2023 and 2024, which represent which year the data was collected in. In the case of (**a**,**e**), a black dashed line displays a statistically significant trend in the data. The trend is monotonically increasing in (**e**), but is monotonically decreasing in (**a**). (**a**) Hot Pixels over the complete dataset. (**b**) Hot Pixels in 2024. (**c**) Boring Pixels over the complete dataset. (**d**) Boring Pixels in 2024. (**e**) Cold Pixels over the complete dataset. (**f**) Cold Pixels in 2024.

Finally, we will observe the number of pixels classified as noise by each of the three pixel isolation algorithms—Hot, Boring, and Cold. These values are displayed in Figure 9. Only panels (**a**) and (**c**) exhibit a significant trend in Figure 9. When normalized for scene in panels (**b**), (**d**), and (**f**) of Figure 9, there are no trends in the pixel counts. The mild trends in the complete dataset and the lack of trends carried into the 2024 data indicate that, across the Hot, Boring, and Cold Pixel types, there was not a strong, consistent trend. In combination with the earlier trends displayed in Figure 7 and Figure 8, we can see that any degradation experienced by the Nadir camera has only existed convincingly in the Nadir RoCo Errors.

## 4. Discussion

### 4.1. Radiation Environment

Radiation for this study is defined as a flux of particles. We are interested in radiation that is either photons (X-ray or gamma ray) or ions or electrons. Sources for these particles are either solar or cosmic. These particles affect either the lenses of the detectors or the instrument electronics. Overall the radiation environment at the space station orbit is on the order of 0.3 nGy/s [20], which is approximately a factor of 3 times the dose obtained at sea level [21].

While radiation hardening within integrated circuits and memory devices has been studied and implemented in a variety of space-based integrated circuits [10,11,12], no such techniques were applied to the DAVIS 240C cameras utilized in the Falcon Neuro payload. Additionally, while physical barriers can be placed around an instrument to protect against radiation effects, radiation shielding due to the presence of a physical aluminum barrier was limited in the Falcon Neuro payload to a quarter inch of 6061 aluminum, with no radiation protection applied to the lens apertures. There are not currently any production EVS chips that utilize radiation hardening of integrated circuits or memory devices.

The effect of radiation on the instrument lenses will be ignored for this study, as the instrument was never used to image objects of small angular resolution, and so no image degradation over time was ever recorded. The reason optical radiation degradation was not measured was because the instrument was mostly used to determine the presence of lightning events and of city flyovers, as well as blob detection of stars and satellites, and none of these requires high resolution imagery.

The radiation effects on the instrument electronics were more pronounced in that the instrument showed signs of bit-flipping leading to Hot Pixels in the instrument. The effect of radiation on instrument electronics is well understood [13,22,23] and we use this information to understand some of what noise was measured by the instrument.

There are two sets of radiation effects to be considered. The first is single-event types of effects. These include single event latchup (SEL), when a charged particle generates a parasitic transistor effect in the instrument electronics. Such an effect can permanently damage the electronics or disrupt the electronics until the instrument is powered off and then on again. Similarly, single event burnout (SEB) occurs when charges produced by incoming radiation initiate an avalanche of current, which in extreme cases can result in the actual melting of the electronic components. Both SEL and SEB result in measurable disruptions in the instrument data and have not been observed on the Falcon Neuro data stream.

A transitory effect on electronics results in a single-event upsets (SEUs). These are expressed as a single bit flip in the memory of the instrument. Results from Neuro show that these upsets are likely taking place, as seen by the occurrence of Hot Pixels. However, the number of Hot Pixels recorded throughout the course of the mission (see Figure 6 and Figure 9) has remained constant. Furthermroe, the study of the Boring Pixels (see Figure 6 and Figure 9) indicates that these pixels also did not change appreciably in time over the course of the mission, and so we conclude that transient radiation effects did not significantly impact the overall performance of the instrument.

The second effect on electronics results in an overall degradation in the ability of the electronics to output signal. Essentially, the gain on the amplifiers needs to increase in order to operate properly. We conclude that radiation effects on the instrument, both transient and chronic, have not had a significant impact on the noise output of the Neuro instrument.

### 4.2. Origin of Noise Types

The origins of noise events in EVS pixels have been studied extensively; however, the precise causes of all noise types are still difficult to determine. In general, a noise event occurs when random variations in the signal entering a pixel’s comparator stage cause deviations from the pixel’s reference level by an amount larger than either threshold in the absence of any true change in the incoming light signal. Functionally, the pixel generates events in response to these changes, and so-called “noise events” are indistinguishable from signal events in the sensor output. Graca and Delbruck showed that noise in the DAVIS sensor used in this study is dominated by photon shot noise [24], and Graca et al. predicted the minimum noise power when the sensor is optimally biased to be 2× the photon shot noise [25]. This, along with the illumination-dependent temporal response of the pixel’s photoreceptor circuit, results in noise event rates that vary significantly as a function of illumination [24,26,27,28].

Shot noise and illumination-dependent bandwidth are a baseline to understand the signal variations that result in noise events. Because the data we have analyzed from Falcon Neuro does not contain node voltages, we are left to interpret these noise event types described here based on underlying assumptions about pixel circuitry, non-idealities, and known noise behavior. With balanced thresholds, noise events tend to occur in pairs of opposite polarity; however, with imbalanced ON and OFF thresholds, overall noise levels can be reduced [29]. This technique was used to bias the Falcon Neuro payload when collecting the data presented here, which has important implications for analyzing the different types of noise presented above and their possible sources.

Hot Pixels are likely a result of a mismatch in comparator thresholds. Due to systematic variations in doping density in CMOS manufacturing processes, thresholds vary between individual transistors. In a DAVIS pixel, two transistors comprise each comparator, and each pixel has an independent comparator for ON and OFF events. Threshold mismatch between transistors can be approximated by a Gaussian distribution, and the 1-sigma mismatch for the device size and manufacturing process is several mV. Typical comparator thresholds used in the DAVIS pixel range from 50 mV up to a few hundred mV If we assume the biases used in this study correspond to an average threshold of ≈50 mV and the 1-sigma mismatch is ≈7 mV, the small fraction of fraction of pixels (0.15%) that are 3 standard deviations more sensitive than the mean will respond when the pixel’s change amplifier output changes by 0.29 mV. This means relatively small signal changes due to shot noise can more easily trigger a noise event in those pixels. When the threshold is sufficiently small relative to the shot noise and mismatch, “hot” pixels corresponding to the “sensitive” tail of the distribution of pixel thresholds begin to fire at a high rate and dominate the noise across the array.

Alternatively, Cold Pixels could correspond to the other tail of the threshold distribution. Using the example above, the least sensitive 0.15% of pixels in the array would not report an event until the log-signal change reached 0.71 mV or greater. In this case, shot noise would be extremely unlikely to cause enough signal deviation to result in a noise event.

The pixel-level behavior that most likely contributes to Boring Pixels is junction leakage (leakage current) in the reset transistor [1]. After a pixel reports an event, its reference level is reset so that the pixel responds to changes around this new “memorized” photocurrent. This reset is accomplished by shorting the input and output nodes of the change amplifier with a single transistor operating as a switch. When the switch is “open”, ideally no current should flow. However, in practice, charge carriers from the substrate can leak across the P-N junction, which gradually decreases the charge on the change amplifier output. In the DAVIS, this gradual discharge reduces the voltage at a steady rate until reaching the ON comparator threshold, at which point an ON event is recorded. These events are referred to as “leak events” and are characterized by periodic ON events [30]. Since this P-N junction is exposed to light, it effectively acts as a photodiode, and the discharge rate increases with illuminance, causing the rate of leak events to increase at higher light intensities. One possible explanation for these Boring Pixels is that in some pixels, the reset transistor may exhibit a higher leak rate than in others. As stated previously, Boring Pixels are often “hot”; however, many Hot Pixels are not boring. This behavior could indicate that most Boring Pixels have a combination of a high leak rate in the reset transistor and a relatively sensitive ON threshold. A pixel can also exhibit boring behavior if transistor mismatch causes one of the thresholds to be below (or extremely close to) 0, so that an event is triggered right after the pixel exits the reset state, resulting in an event rate equal to the inverse of the refractory period.

RoCo errors are the result of the circuits that read out events from individual pixels in the array. Even the most advanced event cameras have finite readout bandwidth, and the way in which events are prioritized to eliminate or reduce data loss and minimize latency is referred to as arbitration. Many different arbitration schemes exist, so the prevalence and nature of RoCo errors will undoubtedly vary between sensor models. In the DAVIS 240C sensors employed in Falcon Neuro, a pixel communicates an event by first transmitting a row request signal along a line that is shared by all pixels in that same row [3]. The arbiter then selects one row from among all rows with an active request and sends an acknowledge signal, at which point all columns in the selected row send a column request signal as the row address is registered and sent off the chip. Next, each column address is read sequentially, such that the row address does not need to be read out separately for pixels in the same row that report events within a short period of time. The exact cause of these RoCo errors has not been studied; however, it is almost certainly related to the fact that the readout circuits share request and acknowledge signals in both the row and column directions.

The final noise category described in this paper as “lonely events” refers to noise events that are not spatio-temporally correlated with any other events. As with other noise types, these events are likely caused by shot-noise variations in the background illumination signal. These variations will trigger events at random times according to a Poisson process. In pixels that do not have abnormally low threshold levels, these events typically occur at relatively low rates.

## 5. Conclusions

The Falcon Neuro payload is an experimental, first-of-its-kind payload that has collected data between 14 January 2022 and 24 September 2024. Falcon Neuro operates two EVS cameras pointed in approximately orthogonal directions. During the time on orbit, the Falcon Neuro payload has been exposed to the space environment. In addition, the payload was moved to a new location aboard the ISS during its mission, heavily affecting the scene in each camera FOV. Due to these changing conditions, our results focus primarily on data taken with the same bias settings, observing only data recorded with Standard Biases, with an analytic preference for the data taken in 2024.

Observation of the data produced by the payload has revealed that there are four distinct types of noise prevalent to the Falcon Neuro EVS cameras. Noise includes: Hot Pixels, Boring Pixels, RoCo Errors, and Lonely Pixels. Additionally, we measured Cold Pixels to observe if pixels were experiencing a reduction in sensitivity over the duration of the mission. Using six distinct algorithms, we isolated and analyzed each of the listed noise types, including Cold Pixels, allowing us to observe meaningful changes that may indicate degradation in the noise of the two EVS cameras over the course of the Falcon Neuro mission. We use a total of 18 measurements to test for trends—12 measurements count the number of events produced by each noise type, 6 for each camera, and 6 measurements count the number of pixels produced by Hot, Boring, and Cold Pixel isolations, with three results for each camera. Once the noise types were isolated and assembled into a time series, a Mann–Keller test for monotonic trends was implemented to detect significant trends in the noise. Using the analytically significant 2024 data, where both scene and bias were normalized, the MK test at the 95% confidence interval revealed two noise categories with statistically significant trends. Both trends were in the Nadir camera and can be seen in Figure 8. Of those two trends, only one persisted through an MK test at the 99% confidence interval, with that noise type being the RoCo Errors. The other significant trend in the Nadir camera—EDF Noise Events—can possibly be explained by other effects, such as high correlation to input data, or a high variance in the EDF Noise Events. However, the RoCo Error trend in the Nadir camera cannot be attributed to other effects and is most likely a symptom of degradation in the Falcon Neuro camera, with radiation being the most likely contributor to the degradation effect.

Given only one category of noise exhibited a strong, consistent trend across the 18 measures of noise types, it is reasonable to conclude that the Falcon Neuro camera system has not experienced significant degradation in noise levels over the course of its mission—with the notable exception of the Nadir RoCo Errors, which did display a real degradation trend, likely caused by a deterioration in the Nadir camera arbiter. This result is highly encouraging for the future of space-based EVS cameras. While more study is needed on a wider variety of camera types and over longer periods, the resilience of the Falcon Neuro payload indicates that the DAVIS 240C EVS cameras used in Falcon Neuro have a high aptitude for space survival and indicate that EVS cameras in general may be well suited to the space environment.

Future work seeks to investigate the internal features allowing for a high resilience in the DAVIS 240C camera, as well as other EVS camera systems. In particular, future work will focus heavily on the Falcon ODIN payload, launched in April 2025, which serves as a successor mission to Falcon Neuro.

## Figures and Tables

**Figure A4:**
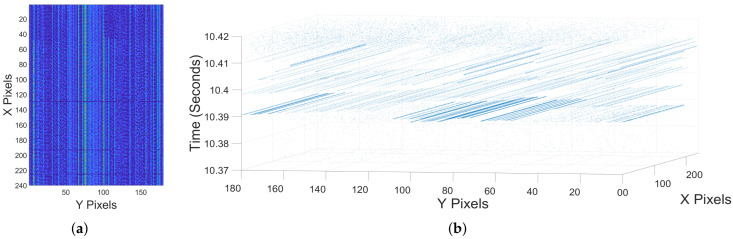
Both panels show the same 50 millisecond window of data. (**a**) A traditional 2D frame of data in which vertical bars are visible representing the RoCo Errors. The color in the image indicates the event intensity, with blue being low intensity and yellow being high intensity (**b**) A 3D image of 50 milliseconds of data, where RoCo Errors can be seen as straight lines along the xy plane occurring in rapid sequence, with full rows recording events within a small window of time.

**Figure A8:**
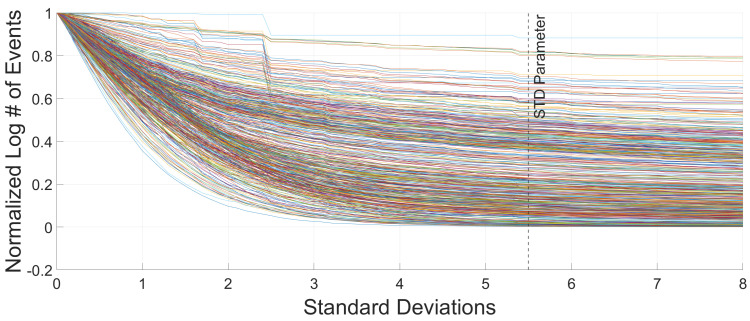
Hot pixel parameter plot showing normalized the log number of events vs standard deviation parameter plot demonstrating how the normalized number of events considered ‘hot’ varies as the standard deviation parameter is changed across all datasets using Standard Biases. Each line represents a single, 60 s dataset. The y-axis represents the normalized number of events (not pixels) that have been considered hot events. Each hot event ultimately belongs to a Hot Pixel. The x-axis represents the standard deviations as used in Equation (1). As the standard deviation increases, the number of hot events tends to decrease. Ultimately, the standard deviation of Equation (2) was set to 5.5, because minor adjustments to the standard deviation parameter produce insignificant changes in the results. Additionally, choosing a standard deviation of 5.5 isolates as many Hot Pixel events as possible while still allowing for minor changes in the parameter and minimizing false positives.

**Figure A10:**
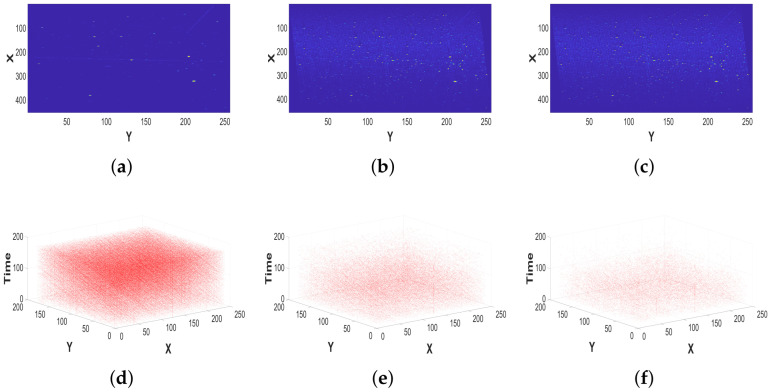
NNb Plot where (**a**–**c**) are three motion-compensated frames from the stars’ supplemental dataset demonstrating how changing the time parameter changes how many visible stars appear. Bright yellow points indicate stars, while the fuzzy blue background largely represents noise. (**d**–**f**) represent the same data as in (**a**–**c**) in a 3D format rather than a 2D format. (**a**,**d**) have a time parameter of c=1000. (**b**,**d**) have a time parameter of c=20,000. (**c**,**d**) have a time parameter of c=30,000. Time is in seconds.

**Figure A13:**
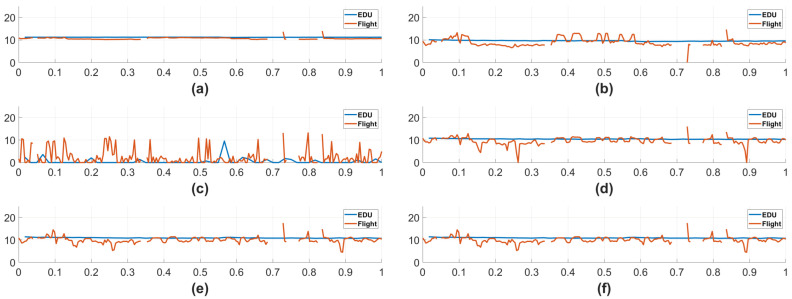
Data from the Ram EDU and Ram Flight Unit cameras using Standard Biases. The Flight Unit data is from 2024, when the scene was normalized. Each plot shows the input data log # of events on the y-axis and the normalized minutes of data on the x-axis. The x-axis is normalized because the EDU only recorded 60 min of data. The EDU data is recorded in blue, and the Flight Unit data is recorded in orange. Each line shows the number of events isolated by each noise algorithm for the EDU and the Flight Unit. We can see that there is a high level of similarity between the EDU and Flight Unit data, suggesting that the Flight Unit has not decayed compared to the EDU. (**a**) Hot Pixels. (**b**) Boring Pixels. (**c**) RoCo Errors. (**d**) NNb Noise Events. (**e**) EDF Noise Events. (**f**) Cold Pixels.

**Figure A14:**
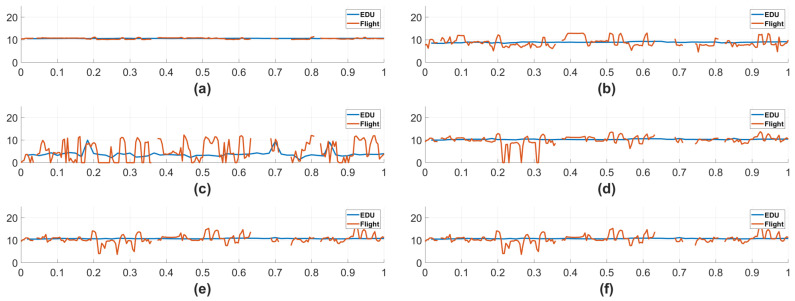
Data from the Nadir EDU and Nadir Flight Unit cameras using Standard Biases. The Flight Unit data is from 2024, when the scene was normalized. Each plot shows the input data log # of events on the y-axis, and the normalized minutes of data on the x-axis. The x-axis is normalized because the EDU only recorded 60 min of data. The EDU data is recorded in blue, and the Flight Unit data is recorded in orange. Each line shows the number of events isolated by each noise algorithm for the EDU and the Flight Unit. We can again see that there is a high level of similarity between the EDU and Flight Unit data, suggesting that the Flight Unit has not decayed compared to the EDU. (**a**) Hot Pixels. (**b**) Boring Pixels. (**c**) RoCo Errors. (**d**) NNb Noise Events. (**e**) EDF Noise Events. (**f**) Cold Pixels.

## Data Availability

The original contributions presented in this study are included in the article/Supplementary Materials. Further inquiries can be directed to the corresponding author.

## Supplementary Materials

The following supporting information can be downloaded at: https://www.mdpi.com/article/10.3390/s25216599/s1, Dataset 1: Ram Stars Set, Dataset 2: Lightning Set, Dataset 3: RoCo Set.

## Abbreviations

The following abbreviations are used in this manuscript:

ISS	International Space Station
EVS	Event-Based Vision Sensor
NNb	Nearest Neighbor
EDF	Event Density Function
ISI	Inter-Spike Interval
AISI	Average Inter-Spike Interval

## Appendix A. Methodology

### Appendix A.1. Three-Dimensional Plots

As a result of the asynchronous design in EVS cameras, it is useful to look at EVS data as a three dimensional volume constituting x-coordinates, y-coordinates, and time in seconds. This shift in perspective is demonstrated in Figure A1. With the new perspective, signal events can be seen as persistent, slanted lines in the three-dimensional image.

The majority of Boring Pixels are also Hot Pixels. This can be seen in a close examination of Figure A2. So, by first removing the Hot Pixels, we can reduce the computational load required to find the remaining Boring Pixels that are not hot.

### Appendix A.2. Parameters

When selecting parameters, three conditions must be met. The first condition is that small changes in the parameter should have a negligible effect on the output results. Second, each parameter should minimize the false positive rate. Third and finally, with the first two conditions taking precedence, parameters should maximize the amount of noise isolated—that is, parameters should minimize the false negative rate.

The factor of 5.5 seen in Equation (1) was selected using the evidence in Figure A8, which shows that the value of 5.5 satisfies the three major parameter requirements.

We have chosen to use a linear decay value of 0.5 because it allows for variations in the linear decay rate while making minimal differences in the results. Similarly, we chose a value of 10 for the change point threshold. A lightning dataset was chosen to compare to demonstrate the low false positive rate, even when observing events that ostensibly have similar behavior to RoCo Errors. The result of the RoCo algorithm isolation can be seen in Figure A5.

For the Falcon Neuro data, we have set c=20,000, with *c* chosen experimentally as seen in Figure A10.

## Appendix B. Results

### Engineering Design Unit Comparison

The Falcon Neuro Engineering Design Unit (EDU) is a copy of the Falcon Neuro flight unit. Comparison data was collected in 2024 from both the Ram and the Nadir cameras mounted on the EDU in order to provide a general comparison to data collected from the orbiting flight unit. Given that we are only interested in noise, EDU data was collected with a lens cap covering the Ram and Nadir EDU optics. In total, 60 min of data was collected from both the EDU Ram and the EDU Nadir cameras, totaling 120 min of comparison data. Given that the EDU has been safely stored in a lab for the duration of the Falcon Neuro mission, there is no expectation of degradation. Therefore, if the number of events for each noise type is similar between the EDU and the flight unit, then we can reasonably assert that the Falcon Neuro flight unit experienced little to no degradation during its mission. We can see in Figure A13 that the EDU and the Flight Unit do indeed record similar values of noise levels across all noise types. Additional spikes in the flight unit data can be largely attributed to variations in scene and additional recording time that the EDU did not have. Similarly, we can see in Figure A14 that the EDU and the Flight Unit are alike, despite the known trend in the Nadir flight unit RoCo errors. The high level of similarity across all noise types and both cameras when comparing the EDU to the flight unit reinforces the evidence that the flight unit has experienced little to no degradation over the course of its mission, despite known exceptions in the Nadir RoCo error event counts and the unusual behavior of the Ram camera Boring Pixel pixel counts.
